# Editorial

**Published:** 1898-03

**Authors:** 


### Editorial.

### NIPPLE-SUCKING IN INFANTS AND YOUNG CHILDREN.

Of the minor bad habits to which infants and young children are addicted, that of nipple-sucking is perhaps the worst.

Many nurses and ill-advised young mothers, in order to quiet a fretting baby, will almost from the hour of its birth, thrust into its mouth a large, soft, collapsible rubber nipple, which comes like the shadow of a great rock in the desolate blank of that period of life.

Babies are creatures of habit, and the rapidity with which they come to consider a rubber nipple essential to their comfort and happiness is almost incredible to one who is not familiar with their peculiarities. It soothes the ruffled temper, stills the thin-edged shrilling, mitigates the pangs of flatulence, and opens the ivory gates of sleep when strenuous pedestrianism on the part of fond, but tired parents has been bald of results.

When the baby flies into the face of nature in attempting transformation of night into day, and assumes the burden of waking at 3 A. M., there is nothing so efficacious in replacing the dislocated natural order of things as the rubber nipple. Some parents whose infant is a confirmed nipple-sucker, in order to be fore-handed, should the baby lose its indispensable companion during the night, keep a supply of "clingfast reversibles" under the pillows, in the bedclothes, conveniently placed on the night-stand, or ingeniously suspended from the ceiling, so that the loss may be instantly made good, at a time when expedition is everything. They are saved the soul-injuring groping, stumbling and blind-feeling in the darkness for the small instrument whose perverted use comes direct from Satan.

The vicious effects of nipple-sucking are not at once apparent, and doting parents imagine that they have found a "sure thing" for making easy the care of babies, but the swift footsteps of the nemesis are close behind them. The first of these ill effects manifests itself in a progressive difficulty on the part of the baby to draw its nourishment from its mother's breast. Accustomed to the large, soft collapsible affair, it does not understand how to shape its lips to the human nipple, and it becomes hopelessly mixed in its innate knowledge of the philosophy of suction. It soon weans itself and no amount of coaxing or ingenuity will win it back, and the mother, though perhaps abundantly able to supply its demands is forced, much to her chagrin, to undertake the herculean task of raising her baby "by hand."

The bad results of the habit unfortunately do not end here. As dentition advances, it will be noticed that the upper teeth begin to protude and that finally the whole upper jaw shows marked deformity, causing a lifetime disfigurement or at least untold worry to the dentist and expense to the parents to correct it.

Besides giving a faulty direction to the teeth and jaw, the presence of the nipple predisposes to mouth-breathing and materially hinders the current of air from entering the lungs. At first the presence of a foreign body in the mouth stimulates the functional activity of the salivary glands, so that there is almost constant drooling. Later on they become accustomed to it, and the saliva is diminished in quantity. This affects stomach digestion remotely and mouth digestion directly. The saliva is thick and gummy; the mouth dry, and the tongue except the part which plays against the nipple is coated, and the breath heavy, especially in the morning, owing to putrefactive changes in the secretions of the mouth. This condition is rendered much worse if the nipple is not kept scrupulously clean and disinfected.

The habit centers around the nipple, but it is apt to broaden out and include other things that can be sucked, the clothing, rags, toys and the like, but strangely enough, never the thumb. A child will often be observed sucking the nipple and at the same time engaged in rubbing itself, sometimes in regions of the body where excitation from friction is apt to inaugurate a habit, more pernicious than the one under consideration.

If no attempt is made to break the child of sucking the nipple, it will in time abandon it voluntarily, but only after the damage accruing from it has been done. Some children persist in it up to the age of seven or eight years, and then grudgingly discontinue it, shamed out of it by parents or playmates.

Now as to the remedy. In the first place, young and inexperienced mothers should be warned against allowing the baby to acquire the habit. They should under no circumstances allow the baby to suck a rubber nipple or a sugar-teat, "between times," but learn or attempt to quiet it in some other manner. Healthy babies do not need it, unhealthy ones are greatly injured by it. But once the habit is fixed, it is imperative to break it up, the earlier the easier. If the infant has reached the age of appreciation of reward and punishment, both of these expedients should be tried. Reward is more efficacious than punishment, for the former gives pleasurable anticipations, and puts the child on his mettle, while the latter, if constantly enforced tends to make him sly and deceitful. Should both fail, more drastic measures are indicated. Throw the nipple away, reserving none as resource if determination falters, and fight it out. It will mean sleepless nights for the parents, but firm resolve will bring victory and save the child from the mortification of carrying a facial disfigurement through life, which may overshadow its happiness and arouse resentment in after years against its parents who will find difficulty in vindicating themselves.

---

### THE TRANSMISSION OF SYPHILIS.

The question of the transmission of syphilis is again provoking much discussion. The point at issue is in regard to the possibility of the disease being transmitted beyond the second generation, and the arguments for and against are many but inconclusive. Transmission to the third generation is interpreted in different ways by various modern writers. Some describe the taint when found in the grandchild as transmission to the second generation; the majority of English-speaking authorities, however, term the disease when twice transmitted transmission to the third generation. This description would appear to be the more correct, as when the disease has been twice transmitted it has existed in three generations.

The opinions held as to syphilis in general and hereditary syphilis in particular diverge to a large and amusing extent. The reports of the numerous researches made and the views of those engaged in elucidating the matter are so conflicting that it is impossible to arrive at a clear conclusion. One well-known English provincial physician has gone so far as gravely to express his belief that the danger attending an attack of syphilis has been altogether exaggerated, and that the disease is no more to be dreaded and no more difficult to cure than an ordinary cold. In regard to the transmission from parent to child, he contends that this is the rarest of all events. On the other hand, there are those who go to the other extreme, and lay to the charge of syphilis almost every known disease. The truth probably lies in the happy medium, and, while it is obviously absurd to designate syphilis as a trifling complaint, yet it is nearly as unwise to endeavor to make it the scapegoat for every disease. That syphilis in civilized countries is not the virulent and destructive disease it was in past days is a notorious fact, but that it can be transmitted is equally as well known. The point in dispute is not in respect to the possibility of its transmission, but as to the number of generations through which the taint may be handed down. Dr. George Ogilvie has recently published a paper reprinted from the *British Journal of Dermatology*, which contains many important facts in relation to this subject. After stating as his opinion that the diagnosis of hereditary syphilis can be made from the patient alone, in infancy as well as in adult life, and after showing that it must be granted once for all that absolutely to exclude acquired syphilis is impossible, he proceeds with these remarks: "On another point, strange as it may sound, it is desirable to come to an agreement, viz., as to what is to be considered hereditary syphilis. That syphilis of the parents, apart from being actually transmitted to the child, may produce in the latter certain pathological states, such as general debility, slow and imperfect development, some backward condition, liability to other diseases, etc., has not escaped the attention of earlier observers. 'Parasyphilis,' as an hereditary condition, is only a new name for an old fact, which, although occasionally doubted or even denied, has on the whole been long recognized. But recently, under the vague term of parasyphilis, all sorts of diseases have been added to the domain of syphilis the syphilitic origin of which is more than doubtful. Whether in the production of such malformations as clubfoot, harelip, congenital amputation, etc., hereditary syphilis plays any part at all, is a question on which a good deal of difference of opinion seems to exist. Those who express themselves in favor of such a connection do so from an impression formed as a result of their personal experience, rather than from a series of well-authenticated cases sufficiently large to establish a clinical fact. Under no circumstances, therefore, can the diagnosis of hereditary syphilis be based upon symptoms of this kind alone, and even when they appear together with unmistakable signs of heredo-syphilis their connection one with the other must remain doubtful."

Many examples of cases of hereditary syphilis collected from all parts of Europe are then given, which, although as evidence they are by no means unanswerable, still present some arguments in favor of transmission to the third generation.

As interesting features of the observations of Dr. E. von During, of Constantinople, who was last year sent on a special commission into the interior of Asia Minor, is that syphilis was not introduced into these parts until about sixty or seventy years ago, so that the living generation would be the third or fourth since the first appearance of the disease.

Another instructive point brought to light in the present discussion on hereditary syphilis is the growing disbelief in Colles' law. The Frenchmen, Fournier and Diday, affirm emphatically that "if a woman bear a syphilitic infant she must of necessity have had the complaint herself." Mr. Jonathan Hutchinson maintains that exception to Colles' law means reinfection. Dr. George Ogilvie is not in agreement with this view, and considers that it has not been proved. Nevertheless, a belief in Colles' law is still more or less an article of faith with the medical profession in England. Dr. Ogilvie is a disbeliever in the theory advanced by many writers, by whom syphilis is credited with exercising a favorable and far-reaching influence by conferring immunity upon generations of descendants without actual transmission of the disease. In relation to this matter he quotes Professor Neumann, of Vienna, who says: "Having made investigations about syphilis in different countries, I am not able to say that I have noticed any perceptible difference in the disease in the various civilized countries." Reference is also made to the investigations recently undertaken in India, in the following words: "Our last experience in India is not such as to strengthen confidence in this theory of national immunization. The havoc which syphilis has made there, among a certain class of population for generations impregnated with the poison, is as bad as or even worse than that caused on virgin soil." From the committee's report it is clear that the malignant and destructive cases were not isolated ones, but were very numerous, and the results so pitiable that "death alone can solve the difficulty."

The case for the transmission of syphilis to the third generation must be regarded as on the whole not proven; the clinical facts adduced in support of the theory do not rest upon a sufficiently solid basis, and have for the most part but an anecdotal value. While it can not be denied that the "dystrophic" influence of syphilis may make itself felt in the third generation, yet until more convincing proof is brought forward to this effect the majority of medical men will remain sceptical. This state of affairs must continue so long as the general practitioner exhibits his present condition of indifference on the subject.—*New York Medical Record.*

